# Intimate Partner Violence Experiences Among Men Living with HIV in Rural Appalachia

**DOI:** 10.1007/s10461-019-02438-3

**Published:** 2019-03-28

**Authors:** Nicole Bryan, Danielle M. Davidov, Taron Dick, John Bassler, Melanie Fisher

**Affiliations:** 1 Section of Infectious Diseases, Department of Internal Medicine, West Virginia University School of Medicine, 2181 Health Sciences Center North, Morgantown, WV 26506-9163 USA; 2 Department of Social and Behavioral Sciences, West Virginia University, Morgantown, WV USA; 3 Department of Biostatistics, West Virginia University, Morgantown, WV USA; 4 Department of Biostatistics, The University of Alabama at Birmingham, Birmingham, AL USA

**Keywords:** Intimate partner violence (IPV), HIV, Rural Appalachia, Revised Conflict Tactics Scale-2

## Abstract

There has been limited study of the syndemic link between HIV and intimate partner violence (IPV) among rural populations in the United States. We utilized the Revised Conflict Tactics Scale-2 to examine the past year prevalence, type (psychological aggression, physical assault, and sexual assault), and the impact of IPV on HIV clinical outcomes among men living with HIV in rural Appalachia. Approximately 39% of participants experienced some type of IPV in the preceding year, with 67% of those individuals experiencing more than 1 type of IPV. Approximately 77% of participants endorsing IPV exposure experienced psychological aggression. Most participants exposed to psychological aggression (70%) and/or physical assault (57%) were both victims and perpetrators, and those experiencing sexual assault reported being exclusively victims (65%). There were no significant differences in clinical outcomes including viral load and CD4 count, which may be secondary to small sample size derived from a clinic population with a high rate of virologic suppression (94%). This study demonstrates the need to assess IPV exposure in men living with HIV and further highlights the intricacies of relationship violence in these individuals.

## Introduction

Intimate partner violence (IPV) refers to a category of domestic violence that according to the Centers for Disease Control and Prevention “includes physical violence, sexual violence, stalking, and psychological aggression” by a spouse or significant other [1]. An increasing body of literature demonstrates a strong association between IPV and short and long term physical and psychological adverse outcomes. In addition to acute injuries and increased risk of mental health concerns, IPV has been linked to central nervous system (CNS) symptoms, gynecological disorders and chronic stress-related conditions, such as gastrointestinal symptoms, viral infections, and cardiovascular problems [2]. Furthermore, there is increasing evidence demonstrating a significant intersection between IPV and the ongoing Human Immunodeficiency Virus (HIV) epidemic [3–6]. For example, women living with HIV who have experienced IPV exhibit higher rates of comorbid disease compared with those who have not experienced IPV [7].

There are multiple points of intersection between IPV and HIV infection including transmission of the virus, clinical outcomes once infection has occurred, and prevention from infection with the use of pre-exposure prophylaxis (PrEP). Numerous studies have demonstrated an increased risk of newly acquiring HIV infection among victims of IPV [8]. The most intuitive risk factor for these individuals is coercive or forced sexual acts. While the overall transmission risk for an individual sexual act is low (estimated at approximately 1 infection per 1000 acts in heterosexual discordant couples [9], repeated events over long periods of time increase the risk of transmission substantially [5]. In addition, IPV of all types is associated with decreased condom use [10–13], which is most likely due to victims’ lack of empowerment to negotiate their use [14]. PrEP, which consists of an oral daily dose of tenofovir–emtricitabine, has emerged as an effective biomedical tool to decrease the risk of acquiring HIV infection in high-risk individuals [15]. Among victims of IPV, data regarding individual interest and willingness to receive PrEP are variable. Some studies indicate victims of IPV are more willing to receive PrEP [16, 17], while others suggest this intervention is less acceptable in this population [18]. Concerns about stigma [19] as well as potential for partner coercion [20] have emerged as potential barriers in this population. Furthermore, there is evidence that African women in serodiscordant heterosexual relationships who are exposed to IPV exhibit lower rates of adherence to PrEP as measured by pill counts, serum tenofovir levels, and self-reported therapy interruptions [21, 22].

While the majority of studies examining IPV as a risk factor for transmission of HIV have focused on female victims, there is also evidence that male perpetrators, particularly those with substance abuse issues, are also at increased risk for acquiring HIV infection. Studies have shown that IPV perpetrators are more likely to engage in unprotected sexual intercourse, have multiple partners, and engage in sexual activity with a partner who uses injection drugs [23–25]. Furthermore, newly acquired HIV infection can also place individuals at risk for IPV as evidence shows that disclosure of a new diagnosis of HIV infection has been identified as a risk factor for intimate partner abuse [3, 26, 27]. A recently published paper by Groves et al. showed that a new HIV diagnosis during pregnancy significantly increased the risk of IPV during the post-partum period among women who had not previously reported victimization in their relationship [28]. It is hypothesized that the introduction of a new HIV diagnosis into a relationship may create additional stress, which may manifest as violence [28]. The stress of a new HIV diagnosis may take several forms including suggestion of infidelity and partner stigma, and exacerbated in the setting of serodiscordant couples or when the partner’s HIV status is unknown [29].

Prior studies have also shown that IPV can negatively impact medication adherence and clinical outcomes in patients once they have acquired HIV infection. Over the past 30 years we have seen tremendous gains in the care of individuals living with HIV. With the advent of highly active antiretroviral therapy (ART), we have now been able to render HIV a chronic disease. Life expectancy of HIV positive individuals in high-income nations now approximates that of the general population [30]. Current ART regimens are highly successful in achieving virologic suppression and maintaining CD4 counts. However, the vast majority of agents must be taken on a daily basis with > 95% adherence in order to achieve these outcomes [31]. Previous work with people living with HIV has demonstrated that stressful life events can negatively affect ART adherence with associated virologic failure and increased risk for HIV-related morbidity and mortality [32–35]. Specifically, IPV has emerged as a major barrier to successful adherence to ART regimens and engagement in clinical care. Studies have shown that victims of IPV are more likely to have CD4 count < 200, detectable viral load, and high no-show rates to clinic appointments [36–39].

The specific mechanisms that may explain why people living with HIV who also experience IPV may have suboptimal engagement in care have only recently been explored [40]. Wingood suggests that for women living with HIV who are exposed to IPV, conflicting prioritizes and complex life challenges (e.g., substance use, poor psychological health, lack of health insurance, and limited access to resources) may interfere with HIV medical care [39, 40]. Furthermore, the inverse relationship between IPV and engagement in protective health behaviors is well-established [40–42], even among men who perpetrate IPV [43]. Lack of social support or isolation may also impact patient’s abilities to obtain HIV services, and power and control dynamics exhibited by some perpetrators of IPV may operate to limit victims’ access to medical care, due to fears of disclosure to medical professionals [44–46].

While the majority of research in this area has focused on women, adverse clinical outcomes have also been observed among gay and bisexual men living with HIV who experience IPV [47, 48]. The lifetime prevalence of IPV (defined as rape, physical violence, and/or stalking by an intimate partner) is 29% among men in the general population (compared with 36% for women) [49]. Taking into account sexual orientation, recent data demonstrate the lifetime IPV prevalence for heterosexual men is 29%, 37% for bisexual men, and 26% for gay men [49]. Among men living with HIV, Siemieniuk et al. reported that 1 in 4 gay and bisexual men engaged in HIV care reported past or present IPV and Ramachandran et al. reported equally high rates of IPV in an urban HIV clinic population, with MSM disclosing higher rates of all types of IPV compared with heterosexual women [47, 48]. Less is known about the specific dynamics of IPV among MSM populations, but there is a growing body of research revealing that many instances of IPV are in fact bi-directional, in which both partners perpetrate violent or aggressive acts against each other [50, 51]. This phenomenon of mutual IPV has been observed in the context of heterosexual and same-sex relationships, including those of people living with HIV [52], which likely reflects additional complexities within this syndemic that have yet to be explored. In response to these findings, many HIV providers are implementing IPV screening protocols within their clinics in an effort to better identify and assist these individuals [53].

People living with HIV and experiencing IPV in rural areas may be at increased risk for adverse clinical outcomes associated with HIV diagnosis, due to the presence of fewer IPV and/or HIV-management resources. Edwards’ critical review of the literature found that IPV perpetrated in rural locations may be more chronic and severe and that physical, psychological, and social health outcomes associated with IPV are worse for rural residents, given disparities in access and availability of IPV services [54]. Long travel times, lack of transportation, physician shortages, and stigma have been cited by rural persons living with HIV as significant barriers to accessing adequate HIV-related care [55]. As a result, individuals living with HIV who have experienced IPV in rural areas may face unique and additional challenges. As such, there is a need to examine the complex interplay between IPV experiences and HIV clinical outcomes in rural populations, with the ultimate goal of designing effective programs to reduce violence and improve management and care for persons living with HIV. However, research examining these issues in a predominantly rural population of people living with HIV in the United States is scarce. The purpose of the present study was to estimate the prevalence of IPV among men living with HIV and seeking care in rural Appalachia over a 1-year period and to examine the relationship between IPV and HIV clinical outcomes among this group.

## Methods

### Setting

This study took place at a Ryan White Positive Health clinic located at a public university in the Appalachian Region of the United States, which cares for over 300 patients. Our clinic serves patients who reside throughout the Appalachian region with the majority of counties designated by the Department of Health and Human Services as “underserved areas” or “underserved populations”. More than 50% of our clinic patients live below the federal poverty level. Ninety percent of the patients are white and seventy-nine percent are male. The majority of the patients acquired HIV in the setting of high-risk sexual contacts with male-to-male sexual contact being the most prevalent at 64% followed by heterosexual exposure (42%). Injection drug use was the next most common risk factor contributing to HIV acquisition occurring in 11.1% of patients. The vast majority of patients (> 99%) are prescribed anti-retroviral therapy and 94% have viral loads < 200 copies/mL.

### Sample and Procedure

Between June 2015 and June 2016, we approached patients from the Ryan White Positive Health Clinic to participate in this cross-sectional cohort study. To be eligible for the study, individuals had to be at least 18 years of age, have a documented diagnosis of HIV infection, and be able to speak and read English. Patients were excluded if they did not meet these criteria, or if they were acutely ill and in need of urgent medical attention, or if they were deemed to be at high risk of psychological trauma as a result of participating in the study. An on-site psychiatrist familiar with each patient who was present in the clinic and independent from the research team identified individuals potentially at high risk of psychological trauma for exclusion.

Potential participants were approached in a private examination room by a trained research team member and invited to participate in the study. Individuals were not approached if there was anyone else in the room, including potential partners, in order to ensure confidentiality and minimize the risk in the event that they were accompanied by a perpetrator of IPV. All participants provided written informed consent as well as a HIPAA waiver prior to completing the survey. The informed consent process included a description of the study and the subject area of the survey as well as data that would be extracted from the medical record, ability of the individual to decline participation with no impact on their medical care, the ability of the participant to opt out of any questions, and the availability of resources for further support if desired. In addition, participants were informed that their survey responses would be anonymous and would not be shared with their care team.

After informed consent was obtained, participants completed a validated written survey about experiences with IPV and a brief demographic questionnaire. Numbered surveys were then collected and placed in a locked collection bin. Medical record numbers of participants were recorded, so the study team could review each consenting patient’s medical record to obtain relevant clinical data. This study protocol was approved by the Institutional Review Board.

### Measures

#### Demographic Data

Demographic information included in the brief questionnaire consisted of age (years), race (White, Black/African American, Asian, native Hawaiian or Pacific Islander, American Indian or Alaska Native, Other), Hispanic/Latino ethnicity (yes/no), education (responses ranging from never attended school to graduate or professional degree), and employment status (employed for wages, self-employed, out of work, homemaker, student, retired, unable to work). Relationship status (married, divorced, separated, widowed, in a relationship and living together, in a relationship but not living together, not in a relationship) at the time of survey administration was also recorded. Participants were also asked to select their estimated annual household income from the following categories: less than $10,000, $10,000–$14,999, $15,000–$19,999, $25,000–$34,999, $35,000–$49,999, $50,000–$74,999, or greater than $75,000.

#### IPV

Exposure to IPV was assessed utilizing the previously validated Revised Conflicts Tactics Scales (CTS-2) [56], which is currently considered the “gold standard” in the assessment of IPV. The survey consists of 78 specific behavioral statements, which are categorized into five scales that include psychological aggression, physical assault, injury, sexual coercion, and negotiation. When completing the survey, participants are asked to specify the number of times each individual behavior occurred over the past year using a 7 point scale ranging from 0 (Never) to 6 (> 20 times). In addition, participants indicate if each behavior happened to them (i.e., victimization) and/or if the behavior was directed at their partner (i.e., perpetration). For this study, we utilized the three subscales that are considered by expert consensus and the survey developers to be the most useful in detecting IPV. These include physical assault, sexual coercion, and severe psychological aggression. Consistent with previous studies [57–60], we considered IPV to be present within the past year if the participant had a score of 1 or higher on the physical assault, sexual coercion, or severe psychological aggression subscales. Surveys that had an overwhelming amount of missing data or were missing data on the IPV questions necessary for calculating scores on the CTS2 scoring rubric, were excluded from analysis.

#### Clinical Variables

In order to evaluate the relationship between IPV and management of HIV disease, engagement in care, and substance abuse patterns for both victims and perpetrators of IPV, we obtained key clinical data from patient medical records. These included participants’ primary risk factor for HIV infection, absolute CD4 counts, and HIV viral load values obtained on each participant for the 1-year duration of the study. HIV risk factors as documented by the primary clinical provider in the participants’ medical record were characterized, and included a history of high-risk sexual behavior involving partners of the same-sex or opposite sex, injection drug use, maternal transmission, or blood transfusion. A viral load of < 200 was considered undetectable to avoid incorporating occasional “blips” that can occur in spite of good adherence to therapy. In addition, a CD4 count of < 200 was considered indicative of advanced HIV/AIDS. Month and year of HIV diagnosis was also noted in order to account for participants who were newly diagnosed during the study timeframe. As an additional indicator of participants’ overall health status, the number of diagnosed co-morbid conditions and the number of hospitalizations during the study period were documented. We also noted the indication for any admissions and whether they were related to the participant’s HIV status.

In order to evaluate patients’ engagement in their HIV care, we examined the overall clinic appointment no-show rate, which was calculated as the number of medical visits not attended or re-scheduled by the participant divided by the total number of scheduled visits over the study period. In addition, given the high rate of prescribed ART in our clinic, we recorded the participants’ overall ART adherence as estimated by their primary HIV provider. ART adherence is regularly assessed in our clinic at every encounter based upon the number of medication doses the patient self-reports that he or she has missed in the previous 30 days [61]. It is documented by each provider in the medical record as an estimated percent with 100% adherence indicating the patient reports missing 0 doses in the previous 30 days. Finally, given the previously reported strong association between IPV and substance abuse among both victims and perpetrators of IPV [25, 39, 47, 62–64], we also documented any tobacco use or ongoing illicit substance use reported by the patients to their providers.

### Statistical Analysis

We analyzed the data using SAS software, Version 9.4 with the significance level set at p < 0.05. Patient characteristics were reported as frequency and percentages for categorical variables and mean ± SD for continuous variables. We performed logistic regression analyses to examine associations between demographic characteristics and experiencing IPV. Associations between experiencing IPV and various binary clinical outcomes were also examined using logistic regression models. For clinical outcomes with greater than two categories, generalized logits models were fit. Each model was adjusted for age and new diagnosis to account for differences in clinical variables, which may be secondary to newly diagnosed HIV infection, such as viral load and CD4 count. Results were reported as odds ratios with 95% confidence intervals.

## Results

### Demographics

From June 2015 to June 2016, 110 surveys were distributed to individuals living with HIV receiving care in the Ryan White clinic and returned for analysis, representing approximately one-third of the total clinic population. A total of five patients were not approached for participation due to concern that they were at high risk for experiencing significant psychological trauma at the time of their clinic visit as determined by the on-site psychiatrist. Six of the returned surveys had an overwhelming amount of missing data and were excluded from analysis. Furthermore, 18 surveys were missing data on the IPV questions necessary for calculating scores on the CTS2 scoring rubric, thus were also excluded from the final data set leaving a total of 86 completed surveys. Of these, 77 surveys were returned by male patients, and ultimately utilized for analysis.

The mean age of participants was 43.7 years old. The majority of participants were White (87.0%), which is not significantly different from the overall clinic population (90.7% White, *p* = 0.483). Over one-third (35.5%) of participants reported a household income of < $15,000 per year and 30.3% of participants had a high school level education or less. The majority of participants (60.9%) reported that they were in a relationship at the time the survey was administered, while 36.2% reported that they were not in a relationship at that time. The majority (83.1%) of survey respondents had acquired HIV infection in the setting of male-to-male sexual contact (compared with 63.5% in the overall clinic population, *p* = 0.011), followed by heterosexual contact (26.0%, compared with 42.1%, *p* = 0.010). These data are summarized in Table 1.

**Table 1:** Association between demographic characteristics and experiencing IPV

Demographic characteristic	Overall		IPV		No IPV		Odds ratio^a^ (95% CI)	p
	n = 77 (% or SD)		n = 30 (% or SD)		n = 47 (% or SD)			
Age (SD)	43.7	(12.7)	40.0	(11.8)	46.0	(12.7)	0.957 (0.918, 0.998)	0.0393
Identify as Hispanic								
Yes	2	(2.6)	0	(0.0)	2	(100.0)	–	–
No	75	(97.4)	30	(40.0)	45	(60.0)	–	–
Race								
White	67	(87.0)	27	(40.3)	40	(59.7)	–	–
Black or African American	4	(5.2)	1	(25.0)	3	(75.0)	–	–
Asian	2	(2.6)	1	(50.0)	1	(50.0)	–	–
American Indian or Alaskan Native	1	(1.3)	0	(0.0)	1	(100.0)	–	–
Other	3	(3.9)	1	(33.3)	2	(66.7)	–	–
Marital status								
Now married	16	(21.9)	4	(25.0)	12	(75.0)	–	–
Widowed/divorced/separated	13	(17.8)	4	(30.8)	9	(69.2)	–	–
I prefer not to answer	1	(1.4)	0	(0.0)	1	(100.0)	–	–
Never married	43	(58.9)	20	(46.5)	23	(53.5)	–	–
Relationship status								
Not in relationship	25	(36.2)	10	(40.0)	15	(60.0)	–	–
In a relationship but not living together	8	(11.6)	2	(25.0)	6	(75.0)	–	–
In a relation and living together	34	(49.3)	13	(38.2)	21	(61.8)	–	–
I prefer not to answer	2	(2.9)	2	(100.0)	0	(0.0)	–	–
Highest education level								
Beyond Bachelor’s Degree	16	(21.0)	4	(25.0)	12	(75.0)	0.602 (0.142, 2.550)	0.4903
Some College or Bachelor’s Degree	37	(48.7	16	(43.2)	21	(56.8)	1.053 (0.350, 3.165)	0.9269
High School Graduate or Less	23	(30.3)	9	(39.1)	14	(60.9)	1.000	–
Current employment status								
Out of work/unable to work	17	(23.3)	8	(47.1)	9	(52.9)	–	–
Employed for wages/self employed	50	(68.5)	19	(38.0)	31	(62.0)	–	–
A home maker	1	(1.4)	0	(0.0)	1	(100.0)	–	–
A student	1	(1.4)	1	(100.0)	0	(0.0)	–	–
Retired	4	(5.5)	0	(0.0)	4	(100.0)	–	–
Household income (all sources)								
Less than $15,000	27	(35.5)	12	(44.4)	15	(55.6)	2.335 (0.208, 26.192)	0.4917
Less than $25,000 ($15,000–$25,000)	10	(13.2)	4	(40.0)	6	(60.0)	2.122 (0.148, 30.472)	0.5799
Less than $50,000 ($25,000–$50,000)	20	(26.3)	6	(30.0)	14	(70.0)	1.155 (0.095, 14.072	0.9098
$50,000 or more	15	(19.7)	6	(40.0)	9	(60.0)	1.736 (0.139, 27.617)	0.6681
I prefer not to answer	4	(5.3)	1	(25.0)	3	(75.0)	1.000	–
HIV risk factor								
Male-to-male sexual contact								
Yes	64	(83.1)	25	(39.1)	39	(60.9)	0.604 (0.155 2.352)	0.4673
No	13	(16.9)	5	(38.5)	8	(61.5)	1.000	–
Heterosexual contact								
Yes	20	(26.0)	7	(35.0)	13	(65.0	1.258 (0.387, 4.087)	0.7027
No	57	(74.0)	23	(40.4)	34	(59.6)	1.000	–
Injection drug use								
Yes	5	(6.5)	2	(40.0)	3	(60.0)	1.347 (0.178, 10.190)	0.7732
No	72	(93.5)	28	(38.9)	44	(61.1)	1.000	–
Blood transfusion								
Yes	2	(2.6)	1	(50.0)	1	(50.0)	1.876 (0.111, 31.851)	0.6632
No	75	(97.4)	29	(38.7)	46	(61.3)	1.000	–

^a^Odds ratios with respect to experiencing IPV, adjusted for age and new diagnosis

### IPV Prevalence

Thirty (39.0%) participants reported exposure to some form of IPV within the previous year, while 47 (61.0%) reported no exposure to IPV. Overall, the demographic characteristics were similar between individuals who reported IPV and those who did not. However, IPV-exposed participants were significantly younger with a mean age of 40.0 years old compared to those who were not exposed who had a mean age of 46.0 years old (p < 0.05). Other than age, no other demographic factors were associated with an increased risk of IPV in our sample (Table 1). Of the individuals not in a relationship at the time of the survey administration, ten (40.0%) reported exposure to IPV in the past year. In addition, there were two individuals who preferred not to disclose their relationship status, both of whom met criteria for IPV exposure within the past year.

### Characterization of IPV

In order to further characterize the experiences of those participants who reported exposure to IPV within the past year, we examined the types of IPV experienced (Table 2). Using the classification system of the CTS2, we divided participant IPV experiences into categories of psychological aggression, physical assault, sexual assault or any combination of these three groups. Of the 30 participants who reported exposure to IPV in the past year, the majority (66.7%) reported experiencing more than 1 category of IPV, including 8 individuals (26.7%) who reported experiencing all three types. Consistent with other studies, psychological aggression was the most common form of IPV reported alone or in combination with other IPV categories (n = 23). Seven participants reported experiencing physical assault and/or sexual assault in the absence of psychological aggression.

**Table 2:** Characterization of types of IPV experienced (n = 30)

	n (%)
Types of individual IPV	
Psychological aggression	6 (20.0)
Physical assault	1 (3.3)
Sexual assault	3 (10.0)
IPV combinations	
Psychological aggression and physical assault	7 (23.3)
Psychological aggression and sexual assault	2 (6.7)
Physical assault and sexual assault	3 (10.0)
Psychological aggression, physical assault, and sexual assault	8 (26.7)

### IPV Perpetration

We also examined the directionality of IPV among participants who reported they had been exposed (Table 3). For each category of IPV, we determined if each respondent was a victim, a perpetrator, or both a victim and a perpetrator of IPV. For both psychological aggression and physical assault, the majority of participants who reported exposure to these forms of IPV were both victims and perpetrators. For respondents who reported exposure to psychological aggression, 16 (69.6%) reported they were both a victim and a perpetrator, compared to 6 (26%) who reported only being a victim and 1 (4.3%) who reported only perpetration. Similar results were noted in respondents who reported instances of physical assault. Among these individuals, 11 (57.9%) reported they were both a victim and a perpetrator, compared to 6 (31.6%) who were victims only and 2 (10.5%) who were perpetrators only. In contrast, of the 16 participants who reported exposure to sexual assault, the majority (68.8%) reported that they were exclusively victims, while the remainder reported they were both perpetrators and victims. No respondents reported being solely a perpetrator of sexual assault.

**Table 3:** Evaluation of IPV experiences of victims and perpetrators among men living with HIV

Type of IPV	Total (%)	Victim	Perpetrator (%)	Both (%)
Psychological aggression	23 (100)	6 (26.0)	1 (4.3)	16 (69.6)
Physical assault	21 (100)	7 (33.3)	2 (9.5)	12 (57.1)
Sexual assault	17 (100)	11 (64.7)	0 (0)	6 (35.3)

### IPV and Clinical Outcomes

Our final aim was to determine if exposure to IPV was associated with clinical outcomes in people living with HIV. There was no significant difference in the number of patients with a detectable viral load, CD4 count < 200, or 100% ART adherence between those who reported exposure to IPV and those who did not (Table 4). While there was an increased percentage of patients with a high no-show rate among those who reported IPV exposure, this difference between groups did not reach significance. Similarly, ongoing illicit drug use was reported by more participants exposed to IPV than non-exposed participants, but this comparison also did not reach statistical significance. Furthermore, there was no significant difference between the two groups with regards to current tobacco use, number of co-morbid conditions, history of other sexually transmitted infections, or number of hospitalizations.

**Table 4:** Association Between IPV and Clinical Outcomes

Clinical outcome	Overall		IPV		No IPV		Odds Ratio^a^ (95% CI)	p
	n = 77	(%)	n = 30	(%)	n = 47	(%)		
Low CD4 count (CD4 < 200)							3.092 (0.644, 14.850)	0.1586
Yes	8	(10.4)	5	(16.7)	3	(6.4)		
No	69	(89.6)	25	(83.3)	44	(93.6)		
Detectable viral load							0.431 (0.113, 1.649)	0.2190
Yes	18	(23.4)	6	(20.0)	12	(25.5)		
No	59	(76.6)	24	(80.0)	35	(74.5)		
100% Adherence^b^							0.493 (0.116, 2.093)	0.3376
Yes	67	(88.2)	25	(83.3)	42	(91.3)		
No	9	(11.8)	5	(16.7)	4	(8.7)		
Hospitalized							1.721 (0.488, 6.064)	0.3984
Yes	14	(18.4)	6	(20.7)	8	(17.0)		
No	62	(81.6)	23	(79.3)	39	(83.0)		
Tobacco use^c^								
Never	18	(23.4)	8	(26.7)	10	(21.3)		
Former	29	(37.7)	9	(30.0)	20	(42.6)	0.783 (0.215, 2.855)	0.7105
Current	30	(39.0)	13	(43.3)	17	(36.2)	0.985 (0.295, 3.290)	0.9804
Illicit drug use							2.155 (0.530, 8.756)	0.2833
Yes	10	(13.0)	6	(20.0)	4	(8.5)		
No	67	(87.0)	24	(80.0)	43	(91.5)		
History of other STI’s							1.010 (0.384, 2.653)	0.9843
Yes	36	(46.8)	15	(50.0)	21	(44.7)		
No	41	(53.2)	15	(50.0)	26	(55.3)		
More than 1 HIV risk factor							1.093 (0.306, 3.902)	0.8910
Yes	14	(18.2)	5	(16.7)	9	(19.2)		
No	63	(81.8)	25	(83.3)	38	(80.8)		
High no show rate (> 33%)							1.689 (0.603, 4.737)	0.3188
Yes	25	(32.9)	13	(43.3)	12	(26.1)		
No	51	(67.1)	17	(56.7)	34	(73.9)		
Number of comorbid conditions							–	–
0	4	(5.8)	1	(3.7)	3	(7.1)		
1	22	(31.9)	10	(37.0)	12	(28.6)		
2	17	(24.6)	9	(33.3)	8	(19.1)		
3	9	(13.0)	2	(7.4)	7	(16.7)		
4	17	(24.6)	5	(18.5)	12	(28.6)		

^a^Odds ratios with respect to having each clinical outcome for patients experiencing IPV relative to patients not experiencing IPV, adjusted for age and new diagnosis

^b^Adjusted for age only

^c^Generalized logits model used with ‘Never’ as the referent category

## Discussion

In this study, we utilized the CTS-2 to examine the extent of IPV exposure in men living with HIV in a rural Appalachian clinic over a 1-year period, as well as the relationship between IPV and control of their HIV infection. We found that more than one-third of participants (39.0%) experienced IPV in the past year. This overall IPV exposure rate is similar to prior studies in persons living with HIV, which utilized different assessment tools [36, 39, 53, 65]. The majority of participants in our study had a predominant HIV risk factor of male-to-male sexual contact. There is an overall paucity of literature examining IPV among same-sex couples in spite of the fact that previous work has shown that these men experience IPV at comparable rates to women [66]. Furthermore, Houston and McKirnan showed that urban gay and bisexual men who are victims of IPV are at increased risk for adverse health outcomes, and for engaging in high-risk behaviors, including unprotected sexual intercourse [65]. While we did not directly assess sexual orientation, our study did reveal that IPV exposure is also a significant issue for men in rural Appalachia living with HIV infection and a history of male-to-male sexual contact. To our knowledge, no prior studies have examined IPV in this population. The implications of this finding on public policy in rural areas are considerable given that resources directed toward victims of IPV are often scarce in these areas, and these men may face additional barriers to accessing them.

Psychological aggression was the most common type of IPV reported by our study participants. However, the majority of participants (66.7%) reported experiencing more than one type of IPV including nearly 26.6% who reported experiencing all three types of violence. These findings are consistent with previous reports of types of IPV experienced by men living with HIV [39, 47, 63]. Although direct comparison is difficult, few studies to date have utilized the CTS2 in the population of people living with HIV. Craft and Serovich employed the CTS2 in their study of gay men living with HIV, which was conducted in an urban setting and also found that psychological aggression was the most commonly experienced type of IPV in that population [67]. However, they did not examine if individual participants were exposed to multiple types of IPV.

In this study, we also found that the majority of participants, who reported experiencing IPV in the form of psychological aggression or physical assault, were both victim and perpetrator within their relationship. Thus, the majority of violence experienced within the intimate relationships of these men was bidirectional. This finding is consistent with previous studies demonstrating that in approximately half of all relationships in which violence occurs both partners are perpetrators. Unfortunately, due to small sample size, and lack of data regarding participants’ partners, we are unable to determine if this bidirectional violence is occurring in the context of heterosexual or same-sex relationships. However, it is worth noting that comprehensive review conducted by Langhinrichsen-Roholing et al. demonstrated that this is true for both heterosexual couples as well as gay, lesbian, or bisexual individuals across multiple sample types [50]. Furthermore, Galvan et al. also showed this to be the case for individuals who are living with HIV infection [52]. This is not to suggest that the severity of the violence perpetrated by both partners is also equal, but that the relationship dynamics may be more complex than previously appreciated.

In contrast to the other forms of IPV, the majority of our study participants who experienced sexual assault in their relationship reported that they were solely victims of this behavior. While there were some individuals exposed to this type of violence who reported that it was bidirectional, no participants indicated they were the sole perpetrator. While these results may be due to participants’ reluctance to report their own perpetration of these behaviors, they do suggest that individuals living with HIV in rural Appalachia are more likely to be victims of this form of IPV rather than perpetrators. This finding is particularly consequential in light of the strong association between IPV and HIV transmission secondary to engagement in high-risk behaviors, including unprotected sexual intercourse.

Unlike previous studies, we did not observe any significant differences in HIV-related clinical outcomes between participants who reported exposure to IPV and those who did not. Most notably, there was not significant difference between patient-reported adherence, low CD4 count or detectable viral load between the two groups. One possible explanation for the disparity in our results compared to prior studies is the low sample size of 85, which limited the power of this study to detect significant differences. Furthermore, the overall high rate of prescribed antiretroviral therapy (99.3%) and virologic suppression (94.0%) among our entire clinic population, which may make detection of significant differences more difficult with our smaller sample size. In addition, due to the sensitive nature of the topic patients with significant psychiatric issues or who were otherwise at risk for experiencing emotional distress as a consequence of completing the survey were excluded. Given the well-established link between IPV and poor mental health outcomes [68], this exclusion may have resulted in an underestimation of the frequency of IPV in our clinic population. Furthermore, exclusion of these individuals may have diminished the impact of IPV on key clinical outcomes in light of the association between common mental health problems and poor ART adherence [69].

In addition, we also did not observe a significant association between IPV and illicit substance use, which differs from numerous prior studies [23, 70–73]. One possibility for this discrepancy is the overall low rate of reported illicit drug use, which was gleaned from the participants’ medical record based upon their reports to their primary HIV providers. By this method, we identified only ten participants with ongoing issues with substance use. While the majority of these individuals [6] did report experiences with IPV in the prior year, it did not reach statistical significance. It is possible that we may have found higher rates of substance use in this population had we examined this issue through anonymous survey due to potential fear of provider stigma. Additional research into this potential association would be warranted.

To date, the majority of studies within the United States examining the association between IPV and HIV have primarily focused on urban populations [4, 7, 48, 62], and may not be generalizable to other populations. Internationally, several investigations with people living with HIV in rural areas have revealed high rates of IPV among this population [74–78]. However, the clinical challenges and cultural demographics of patients in these countries are distinct from rural populations in high-income countries. This makes it difficult to apply the findings from these studies to rural areas within the United States. While there are few data on individuals living with HIV, there is evidence that IPV occurs more frequently within relationships and with greater severity in rural communities compared to non-rural locales within the United States [79]. Unfortunately, in spite of this disparity, there are fewer IPV resources available in rural areas, which likely results in worse psychosocial and physical health outcomes for victims who reside there [54, 79]. Residents of the Appalachian region of the country, which possesses one of the largest rural populations in the US, are particularly vulnerable to adverse outcomes. These communities have higher levels of economic distress and have long suffered an extreme shortage of health care providers and services [80]. Furthermore, Davidov et al. recently demonstrated that hospitalizations for IPV were disproportionately higher in Appalachian counties, compared to non-Appalachian areas, suggesting IPV is a significant healthcare disparity issue in this region [81].

Although routine screening for IPV in healthcare settings has not demonstrated effectiveness at reducing violence, improving health outcomes, or improving patients’ quality of life, most medical and advocacy organizations, including those focused on HIV care, recommend IPV assessment and counseling [82–84]. Given the high prevalence of IPV among individuals engaged in HIV care, including the high prevalence of past-year IPV reported in the current study, HIV providers should be equipped to inquire about IPV and provide resources and referral information in a confidential and supportive manner. Ramachandran et al. recommend that IPV inquiry be conducted simultaneously with HIV counseling and testing, and that these processes occur before HIV status is disclosed to patients’ partners, if possible [48, 83]. Clinicians and medical staff providing HIV services should acknowledge the challenges associated with prolonged engagement in medical care IPV victims and should recognize that these barriers (e.g., fears about stigma, loss of confidentiality, and lack of resources) may be exacerbated for patients in rural areas [54]. Cross-collaborations between organizations involved in HIV prevention and medical management and IPV service providers are important to ensure that HIV care delivery is sensitized to those at-risk for or experiencing IPV.

There are a number of limitations to the current study that should be considered in the interpretations of our findings. First, this is a cross-sectional study, and therefore our ability to make conclusions regarding causality or temporal relationships is limited. Furthermore, our design may have introduced selection bias, particularly given the exclusion of 5 individuals due to concern that their participation may have caused them significant psychological trauma, and the need to exclude surveys due to an overwhelming amount of missing data or to the inability to accurately score them. In addition, data analysis is limited by an overall small sample size, which prevented us from being able to adjust for multiple confounders such as substance use, or assess the association between different types of IPV and clinical outcomes. Furthermore, the small sample size in addition to the high rates of prescribed ART and viral suppression, and exclusion of individuals with high risk of experiencing significant psychological trauma likely limited our ability to detect significant differences in clinical outcomes between patients who experienced IPV, and those who did not. An additional limitation to the current study is the lack of data regarding participants’ partners, including their gender, which limits our ability to assess the types of relationships in which different patterns of violence occurred.

To the best of our knowledge, only one other study has examined the issue of IPV in individuals living with HIV in the Appalachian region of the United States [36]. This study was conducted in rural Virginia, and was conducted using in-person interviews to assess for IPV, rather than the revised CTS-2. While participants in this study were from Appalachia, the sample’s demographic profile was substantially different than what we report here, as it included larger percentages of African American and female participants. In addition, unlike the current study, data regarding types of IPV and perpetration were not obtained. Thus, the current study provides additional insight into the IPV experiences of Appalachian men living with HIV. Furthermore, the current study examining relationship dynamics in people living with HIV is particularly relevant given the recent HIV outbreak in West Virginia [85]. Future studies examining the impact of IPV on the transmission of HIV and other STIs, the relationship between different forms of IPV and HIV clinical outcomes, as well as substance use, and the influence of same-sex vs heterosexual relationships on the experience and perpetration of IPV should be pursued.

## Conclusion

Exposure to intimate partner violence is common men living with HIV who reside in rural Appalachia, and is often bidirectional and consists of multiple forms of violence. These nuances should be taken into account when assessing for IPV exposure in these patients.
